# Monitoring of shallow groundwater in Lower Saxony, Germany—spatial variability of redox conditions and benefit of the redox proxy ∆_Mn-Fe_

**DOI:** 10.1007/s10661-025-14557-7

**Published:** 2025-09-10

**Authors:** K. Hamer, J. Ritter

**Affiliations:** Department of Geosciences, University of Bremen, Bremen, Germany

**Keywords:** Groundwater monitoring, Redox conditions, Scale effects, Water framework directive, Groundwater assessment

## Abstract

Surveillance monitoring of shallow groundwater revealed that redox conditions can vary on a small scale. Therefore, the aim of this study was to categorize redox conditions in the groundwater of Lower Saxony, Germany, and to analyze the spatial distribution and trends of parameters related to redox conditions during surveillance monitoring from 1957 to 2015 in Lower Saxony, Germany. Methodically, trends were considered by applying the Mann-Kendall test and redox conditions of groundwater were classified according to the scheme of Jurgens et al. (2009). While the porous aquifers were dominated by anoxic redox conditions, the karst and joint aquifers presented a high percentage of the oxic redox category. A third of the monitoring wells showed trends with respect to redox conditions. Positive ΔMn-Fe values, indicating manganese-reducing conditions, were observed in 17% of the samples, which were mostly taken in areas of high groundwater recharge. Remarkably, different redox regimes were sampled in close neighborhoods within areas that were assumed to be homogeneous with respect to recharge rate, usage, and hydrogeology. In conclusion, parameters that are sensitive to redox conditions should be investigated stepwise; first, monitoring wells should be categorized according to the redox conditions and second, only wells with the same redox conditions should be compared. Third, the parameter ∆Mn-Fe should serve as a proxy for potential changes of the redoxcline, e.g., due to nitrate emissions to shallow groundwater. This stepwise procedure allows the assessment of groundwater and can serve as a basis for the design of measures to reach environmental aims according to the European Water Framework Directive.

## Introduction: motivation and questions

The chemical composition of groundwater is of vital interest because groundwater is a resource for drinking water, and accordingly, groundwater is monitored nationwide and concisely (e.g., McMahon & Chapelle, 2008). In the European Union, such national monitoring results are reported regularly and serve as a tool to assess groundwater composition and develop measures for its sustainable use (2000 /60/EC; Ortmeyer et al., 2022).

The composition of groundwater results from the input of dissolved substances via infiltration and chemical processes into the overlying soil and the aquifer. Other factors influencing the composition of groundwater are the groundwater recharge rate (McMahon & Chapelle, 2008; Tesoriero et al., 2015), land use (Knoll et al., 2019; Kubier et al., 2019), geological heterogeneity (Hansen et al., 2014; Tesoriero et al., 2015), screen length, depth to the groundwater surface of monitoring wells (McMahon & Chapelle, 2008; Wriedt et al., 2019), and redox conditions (Collins et al., 2025). These redox conditions are the result of a succession of electron-accepting processes involving either degrading organic matter or oxidizing minerals such as pyrite. These processes form a sequence of redox zones from oxygenated near the groundwater surface to more reducing conditions (e.g., Appelo & Postma, 2005; Christensen et al., 2000; Su et al., 2018).

The scale of such redox zones ranges from millimeters in fine-grained sediments (e.g., Hansen et al., 2008) to meters and decametres in groundwater recharge areas (e.g., Christensen et al., 2000; McMahon & Chapelle, 2008; Su et al., 2018) and kilometers in confined aquifers (Lovley & Goodwin, 1988). The orientation of the redox sequence in an aquifer can have vertical as well as horizontal components due to different hydraulic conditions in recharge and transition areas (Merz et al., 2009).

This complex combination of factors influencing the composition of groundwater led to the idea of applying mathematical and statistical tools to simplify the picture and to determine proxies or equations to calculate the probable appearance of redox conditions (e.g., Hansen et al., 2008 and 2014) or redox-sensitive parameters such as nitrate (e.g., Knoll et al., 2019 and 2020; Wolters et al., 2022). Accordingly, Wriedt et al. (2019) calculated semivariograms and applied kriging to describe the spatial distribution of nitrate in the shallow groundwater of Lower Saxony, Germany. Further statistical methods, such as principal component analysis (Mouser et al., 2005; Wriedt & Randt, 2019) and machine learning systems (Knoll et al., 2019 and 2020; Erickson et al., 2021), have been applied to find correlations or proxies among parameters and to construct maps describing probable redox conditions or concentrations of parameters that depend on redox conditions, such as nitrate or arsenic, in groundwater. Schafmeister et al. (2023) discussed problems of interpolating concentrations in groundwater spatially and applied Voronoi tessellation and characterized hydrogeological units by a single probable concentration or a range of concentrations so that a mosaic-like picture of spatial units with different concentrations appeared instead of a map with smooth contour lines representing interpolated concentrations. Additionally, such redox conditions can vary spatially and show temporal variability (e.g., Collins et al., 2025).

Against this background, it is a complicated task to regionalize chemical data of groundwater, especially if components are consumed, such as nitrate due to denitrification, or seem to be produced, such as manganese, due to the reduction of manganese oxides in the aquifer. However, Eschenbach et al. (2018) reported good agreement between measured and modeled nitrate in the groundwater of Lower Saxony. Methodically, Eschenbach et al. considered nitrogen in groundwater as the analyzed sum of nitrate and nitrogen from the denitrification of nitrate following the method of Blicher-Mathiesen et al. (1998).

Wolters et al. (2022) applied redox-sensitive species of groundwater composition to classify and rank monitoring wells and to calculate the effects of denitrification in shallow groundwater. Wolters et al. checked the plausibility of their ranking and calculated the effects of denitrification by comparing those results with analysis via the nitrogen-argon method. The authors concluded that their approach allowed to explain the observed differences in denitrification at regional scales.

Another interesting approach that addresses the regionalization of point information from monitoring wells in shallow aquifers is to categorize such wells in an existing monitoring network according to redox categories (Jurgens et al., 2009). This redox classification was recently complemented by a new redox proxy, ∆_Mn-Fe_ (Hamer et al., 2020). As the mathematical difference between the manganese (Mn^2+^) and iron (Fe^2+^) concentrations, the ∆_Mn-Fe_ becomes positive under manganese-reducing redox conditions, especially if nitrate (NO_3_^−^) is present simultaneously and then depletes Fe^2+^ due to oxidation. Consequently, this parameter, ∆_Mn-Fe_ reveals two advantages. First, it identifies monitoring wells with screens in a redox environment where NO_3_^−^ emissions might change the composition of groundwater and, second, in contrast to additional analyses such as the nitrogen-argon method, ∆_Mn-Fe_ can be applied retroactively to long-term data sets because Mn^2+^and Fe^2+^ have been part of routine monitoring programs for decades.

The central question of this paper is how to consider redox conditions varying over short distances in an existing monitoring network. Consequently, the question rises how a monitoring network is capable to describe spatial distribution of redox-sensitive parameters such as NO_3_^−^, Mn^2+^, and Fe^2+^ in shallow groundwater, which is relevant if threshold values are exceeded, and measures to protect and improve groundwater should be verified. Additionally, this study aimed to test whether ∆_Mn-Fe_ is helpful for identifying such areas more easily and observing and analyzing trends.

## Study area

**Fig. 1:**
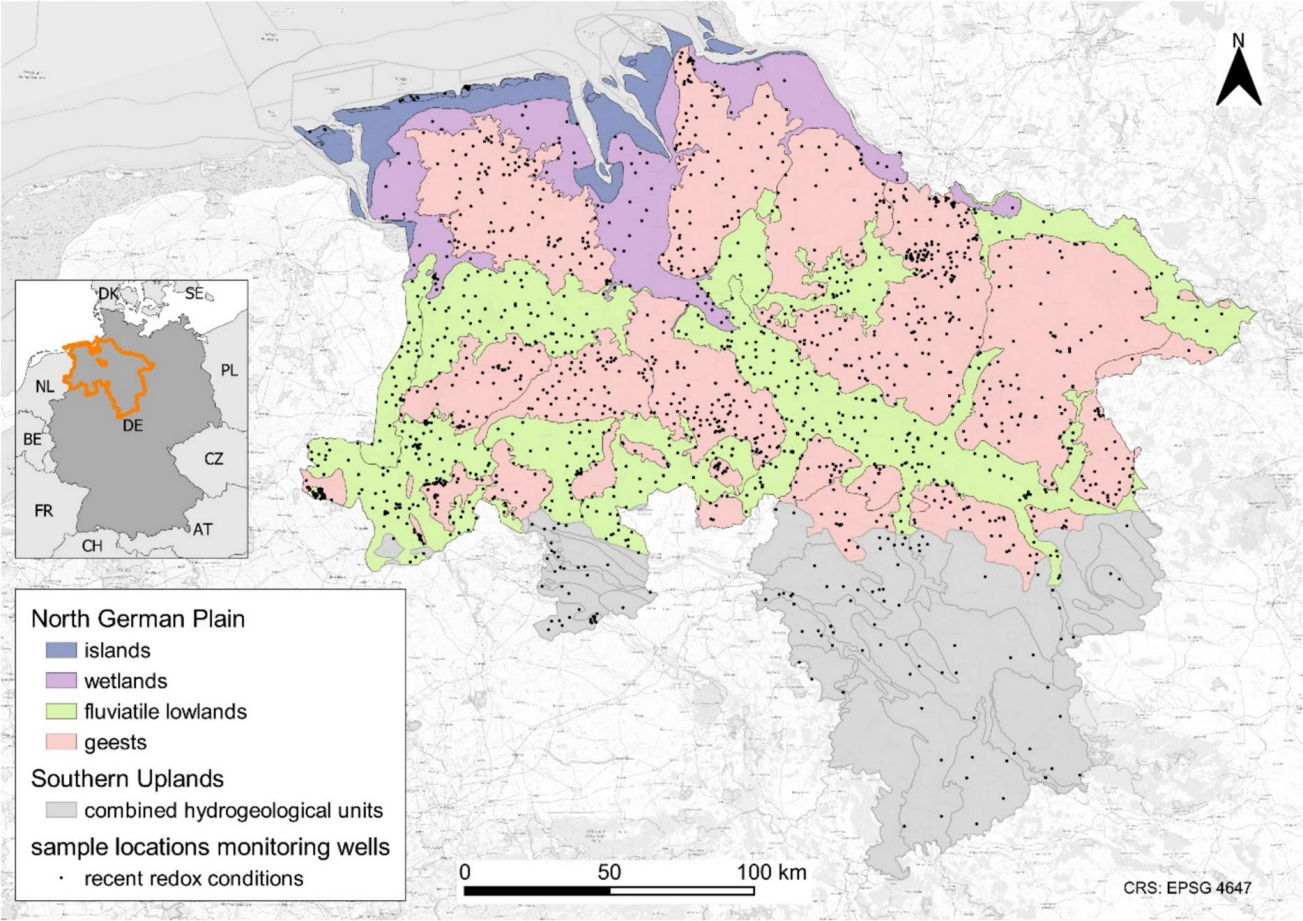
Study area with sample locations and hydrogeological units of the North German Plain and the Southern Uplands (modified from Elbracht et al. (2016))

The study area comprises the federal states of Lower Saxony and Bremen, Germany, and covers approximately 48,000 km^2^ (Fig. 1). The northern part belongs to the North German Plain, which is characterized by a moderate climate with a mean temperature of 9.6 °C and a precipitation of 787 mm/a between 1990 and 2010, whereas in the south a more distinct relief of average mountains in the Southern Uplands results in higher precipitation, partly exceeding 1200 mm/a. In the North German Plain, Cenozoic sediments, which were deposited mainly during the Pleistocene and Holocene, built porous aquifers. Toward the Southern Uplands, these Cenozoic sediments thin out, and Mesozoic and Paleozoic rocks enabled the evolution of karst and joint aquifers, mainly within Mesozoic sand- and limestones. According to their geological characteristics, the Southern uplands were divided into the hydrogeological units Nordwestdeutsches Bergland, Sandmünsterland, Mitteldeutsches Grundgebirge, Thüringische Senke, Münsterländer Kreidebecken, and Subherzyne Senke. The northern part can be separated into four hydrogeological units: islands, wetlands, geests, and fluviatile lowlands. While islands and wetlands were formed predominantly by the North Sea, the lowlands developed along rivers and creeks, and the geests, which were deposited during the Pleistocene, mainly consist of glaciofluvial sediments and tills. In the study area, the mean recharge rate for groundwater is 150 mm/a. The geests feature a moderate relief, with a mean groundwater recharge rate of 200 mm/a, and shallow aquifers, with hydraulic conductivity between 10^–5^ and 10^–3^ m/s, often used as catchment areas for water supply. Fluviatile lowlands and wetlands show a more subdued relief, high water tables, and mean groundwater recharge rates of 128 mm/a and 28 mm/a, respectively (Ertl et al., 2019). All hydrogeological units can be further divided into subunits (Elbracht et al., 2016).

The chemical composition of the shallow groundwater of the northern part is of the alkaline-earth type, which is dominated by bicarbonate or sulfate and chloride. Specifically, on the islands, groundwater occurs as the bicarbonate-predominated alkaline-earth type, whereas groundwater in the wetlands is mainly iron- and sulfate-reducing and belongs to the group of alkaline waters with decreasing bicarbonate contents. Groundwater in the lowlands and the geests is predominantly of the alkaline-earth type, with lower bicarbonate contents. In the Southern Uplands, shallow groundwater is of the bicarbonate-sulfatic alkaline-earth type (Kubier et al., 2019).

Land use is dominated by arable land covering 64% of the area, followed by 24% forests, 10% used for residential purposes and infrastructure, and 1% wetlands and water bodies (Bundesamt für Kartographie & Geodäsie BKG, 2018).

## Data and methods

The data set consisted of ~ 32,000 groundwater measurements from ~ 5300 monitoring wells sampled by the responsible authorities of Lower Saxony and Bremen between 1957 and 2015 (Table 1). Groundwater was sampled after the field parameters of temperature, electric conductivity, pH value, and oxygen content were constant. The samples were filtered through 0.45-µm membrane filters and separated into subsamples. Subsamples for cation analyses were acidified to a pH value < 2 with nitric acid, whereas Subsamples for anion analyses remained nonacidified. Analytical techniques were applied, which offered a detection limit of less than 30% of respective threshold values or criteria of interest.

**Table 1:** Monitoring wells and measurements in the study area (1957–2015)

No	Description of data set	Number of monitoring wells	Number of samples
1	Complete data set 1957–2015	5268	31,946
2	Part of data set (1) with wells and samples including all parameter necessary for redox classification	3914	21,369
3	Part of data set (2) with wells and samples for redox classification of recent time	1872	1872
4	Part of data set (3) for trend tests (including 4 samples or more)	1024	16,072
5	Part of data set (4) for trend tests on monitoring wells with minimum one value of ∆_Mn-Fe_ > 0	348	3002

During the decades of monitoring, the demands for precision and quality of chemical analysis have developed with upcoming analytical options and progress in toxicology. Over this time span, analytical techniques were applied, which offered a detection limit of less than 30% of respective threshold values or criteria of interest. This was the legal requirement according to the European Groundwater Directive (2006/118/EU) and was implemented into German legislation. Since more than 5000 wells were sampled and analyzed regularly, many certified laboratories were involved in the monitoring process and sometimes they applied different analytical techniques. The standard requirements for any analytical procedures were laid down in DIN EN ISO/IEC 17025 (DIN 2018). Recently, the laboratories analyzed anions with ion chromatography or spectrophotometry, and main cations with ICP-OES and trace metals with ICP-MS. Alkalinity was analyzed with a titration (Gran et al., 1981).

### Data management and statistical analysis and tests

The data set was converted into a SQLite3 database. Further analyses were carried out with Python in Spyder and QGIS 3.16.6. The statistical analysis of the hydrogeochemical data was carried out with non-parametric tests and descriptive statistics (Helsel et al., 2020; Reimann & Filzmoser, 2000; Yue et al., 2002). All hypothesis testing was performed at a significance level of *α* = 0.05. In boxplots, the box represented the lower and upper quartiles, with a line representing the median of the data. The whiskers were the upper or lower quartiles plus or minus the 1.5*interquartile range or the minimum or maximum value of the data (Hunter, 2007). The Wilcoxon rank sum test was used to test for differences between two independent groups (Helsel et al., 2020). The Kruskal-Wallis test allowed to test for similarity of distributions of multiple groups (Helsel et al., 2020; Kruskal & Wallis, 1952). Both non-parametric tests were carried out with SciPy in Python (Virtanen et al., 2020).

The Mann-Kendall trend test (Kendall, 1975; Mann, 1945), available in Python as a package by Hussain and Mahmud (2019), was applied to the concentrations of oxygen (O_2_), NO_3_-N, Mn^2+^, Fe^2+^, and sulfate (SO_4_^2−^) to analyze trends. The test was performed if the time series included at least four measurements and if the youngest sample was collected after 2003.

### Redox classification and Mn-Fe difference (∆_Mn-Fe_)

The redox classification considers the concentrations of O_2_, NO_3_^−^, Mn^2+^, Fe^2+^, and SO_4_^2−^ to assign redox categories and redox processes (Jurgens et al., 2009) (Table 2). This study excluded samples if anyone of the five parameters was not analyzed. The redox processes were labeled according to the chemical compounds characterizing the redox zone. Mixed processes led to a naming convention, where the respective redox processes were connected by a hyphen (see examples in Table 2).

**Table 2:** Main redox categories and their respective processes acc. to Jurgens et al. (2009)

Redox category	Redox process^a^	Criteria to define process from water quality data [mg/L]				
		O_2_	NO_3_^−^-N	Mn^2+^	Fe^2+^	SO_4_^2−^
Oxic	Oxygen reduction: O2	> 0.5	-	≤ 0.05	≤ 0.1	-
Suboxic	Suboxic	≤ 0.5	≤ 0.5	≤ 0.05	≤ 0.1	-
Anoxic	Nitrate reduction: NO3	≤ 0.5	> 0.5	≤ 0.05	≤ 0.1	-
Anoxic	Manganese reduction: Mn(IV)	≤ 0.5	≤ 0.5	> 0.05	≤ 0.1	-
Anoxic	Iron and/or sulfate reduction: Fe(III)/SO4	≤ 0.5	≤ 0.5	-	> 0.1	> 0.5
Anoxic	Methanogenesis: CH4gen	≤ 0.5	≤ 0.5	-	> 0.1	≤ 0.5
Mixed (oxic-anoxic)	Mixed oxygen, nitrate and manganese reduction: O2-Mn(IV)	> 0.5	-	> 0.05	≤ 0.1	-
Mixed (oxic-anoxic)	Mixed oxygen, nitrate, manganese, iron and sulfate reduction: O2-Fe(III)/SO4	> 0.5	≤ 0.5	-	> 0.1	> 0.5
Mixed (anoxic)	Mixed nitrate and manganese reduction: NO3-Mn(IV)	≤ 0.5	> 0.5	> 0.05	≤ 0.1	-

^a^The abbreviations for dominant redox processes according to Jurgens et al. (2009) are specified, e.g., “NO3-Mn(IV)” is the short term for the occurrence of denitrification and reduction of manganese

Samples were excluded from categorization if the detection limit of at least one redox-sensitive parameter exceeded the threshold value of the redox classification. For further statistical analysis, concentrations that were below the limit of detection were considered the value of the detection limit. The latest samples from any monitoring well between 2003 and 2015 were considered the recent redox conditions in the study area.

The parameter ∆_Mn-Fe_ is defined as the difference between Mn^2+^ and Fe^2+^ concentrations in mg/L (∆_Mn-Fe_ = Mn^2+^ [mg/L] − Fe^2+^ [mg/L]).

The screen depth below groundwater was considered the depth of the screen center below the groundwater surface. Forty-seven percent of the monitoring wells that described the recent conditions were 2 m long, and 83% were up to 5 m long (Table 3).

**Table 3:** Screen length [m] and screen depth below the water table [m] of the monitoring wells

				Screen length [m]				Screen depth below groundwater [m]			
		Hydrogeological unit		*n*^a^	Q_1_^b^	Q_2_	Q_3_	*n*^a^	Q_1_	Q_2_	Q_3_
North German Plain		Islands		46	2	2	2	40	6	11.6	20.0
		Wetlands		82	2	2	3	60	11.8	17.3	24.7
		Fluviatile lowlands		676	1.9	2	3	474	6.9	13.3	27.2
		Geests		974	2	2	3	653	5.6	12.6	23.6
Southern Uplands		Nordwestdt. Bergland		70	2	4	7	43	4.3	7.4	12.8
		Sandmünsterland		11	1.5	2	2.5	9	6.6	7.9	14.4
		Mitteldt. Grundgebirge		3	-^c^	-^c^	-^c^	0	-^c^	-^c^	-^c^
		Thüringische Senke		6	7	14	22.5	1	-^c^	-^c^	-^c^
		Münsterländer Kreidebecken		3	-^c^	-^c^	-^c^	3	-^c^	-^c^	-^c^
		Subherzyne Senke		1	-^c^	-^c^	-^c^	0	-^c^	-^c^	-^c^
sum				1,872	2	2	3	1,283	6.1	12.8	24.1

^a^*n*: number of wells

^b^Q_*n*_: *n*^th^ quartile

^c^-: no data available

## Results

**Fig. 2:**
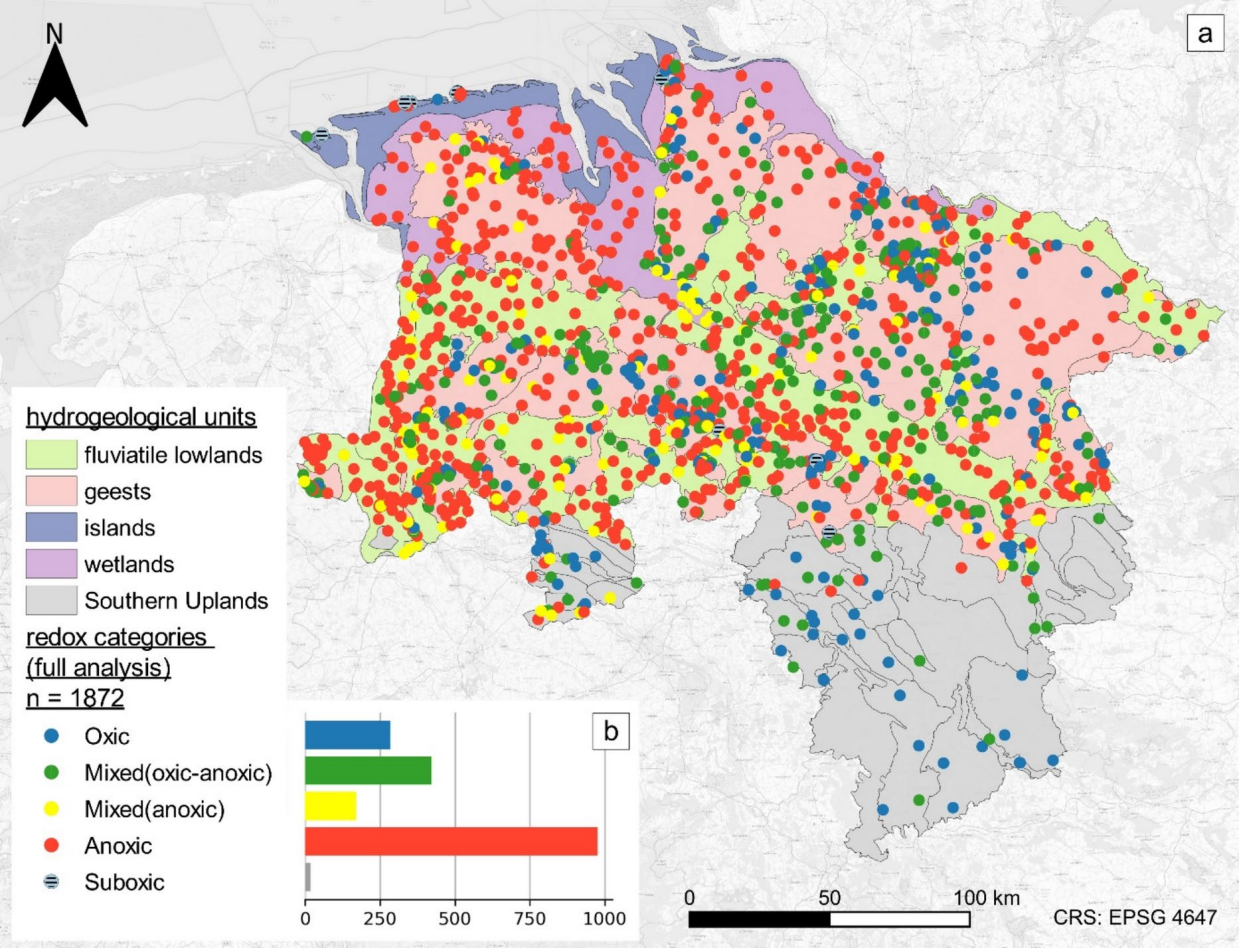
Recent redox conditions in shallow groundwater. The anoxic category is the most common (**b**), but its spatial distribution depends on the characteristics of the hydrogeological units (**a**)

At least 1872 monitoring wells represented the recent redox conditions of shallow groundwater in the study area. The spatial distribution of the monitoring wells revealed that most samples were taken from porous aquifers of the North German Plain (95%), whereas the remaining 5% originated from the karst and joint aquifers of the Southern Uplands (Fig. 2a). Within the North German Plains, the hydrogeological units geests and lowlands were sampled most intensely, containing 974 (52%) and 676 (36%) monitoring wells, respectively.

**Fig. 3:**
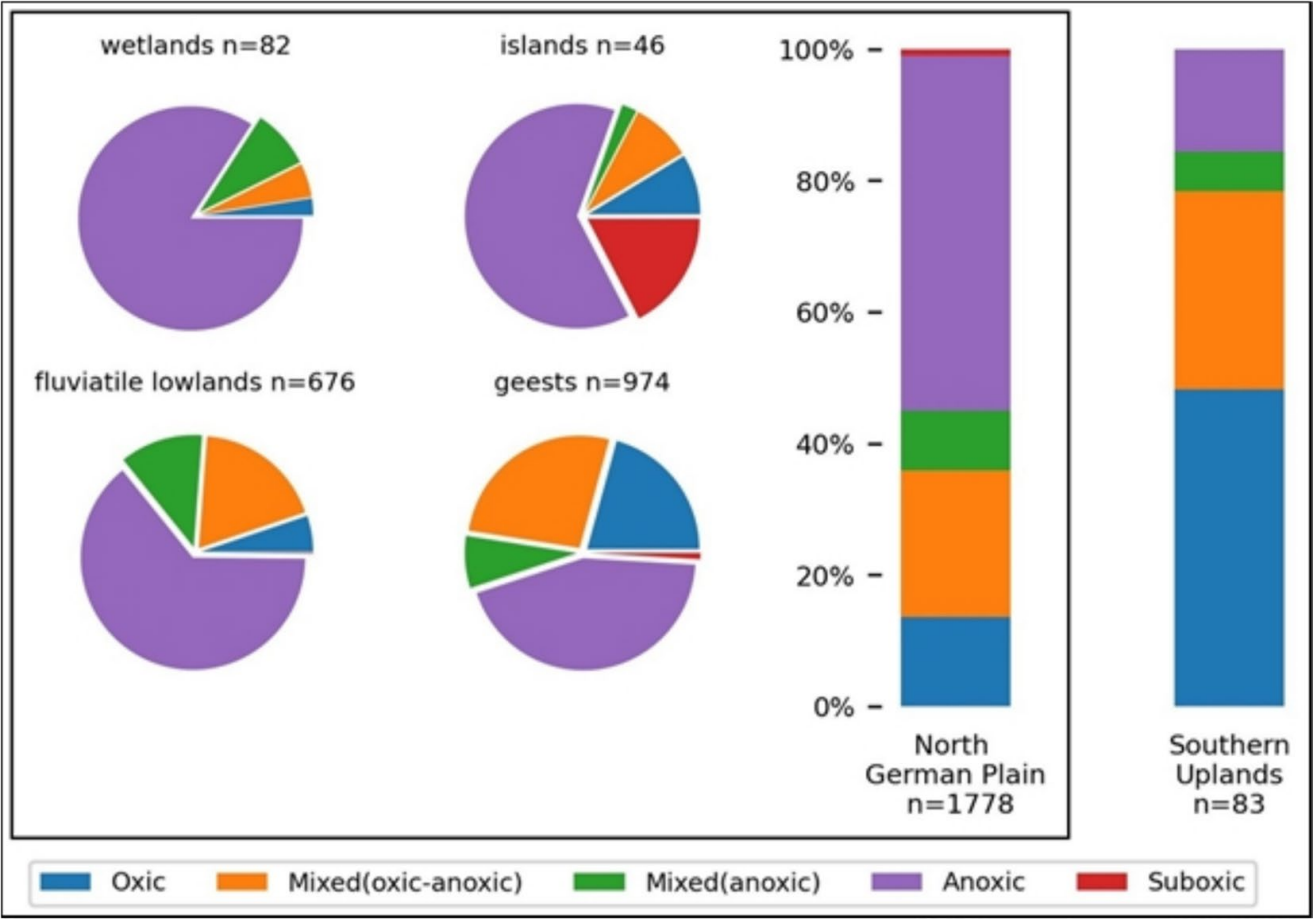
Redox categories in hydrogeological units of the North German Plain and the Southern Uplands. The bar charts represent the summarized regions of this study, while the pie charts are the hydrogeological units of the North German Plain (n: number of monitoring wells)

Most wells in the study area were in an anoxic state (52%). The subsequent redox categories were 23% mixed (oxic-anoxic), 15% oxic, 9% mixed (anoxic), and 1% suboxic (Fig. 2b). The occurrence of redox conditions differed regionally: while the porous aquifers of the North German Plain were dominated by anoxic redox conditions (54%), the karst and joint aquifers of the Southern Uplands showed a higher percentage of the oxic (48%) redox category (Fig. 3, bar charts). The hydrogeological units of the North German Plain revealed a more distinct differentiation of general redox conditions between different hydrogeological settings (Fig. 3, pie charts). The groundwater of the wetlands presented the highest proportion of anoxic (84%) redox conditions. The islands were the only unit where suboxic (17%) groundwater was sampled at a significant level while also being dominated by anoxic conditions. The fluviatile lowlands and geests contained a relatively high proportion of mixed (oxic-anoxic) groundwater, with 19 and 27%, respectively. Likewise, both hydrogeological units differed in their proportions of anoxic and oxic redox conditions. While the monitoring wells from the fluviatile lowlands predominantly had anoxic groundwater (64%), the geests had less anoxic groundwater and showed a greater span of redox conditions ranging from oxic (21%) to anoxic (44%).

### Parameter Δ_Mn-Fe_

**Fig. 4:**
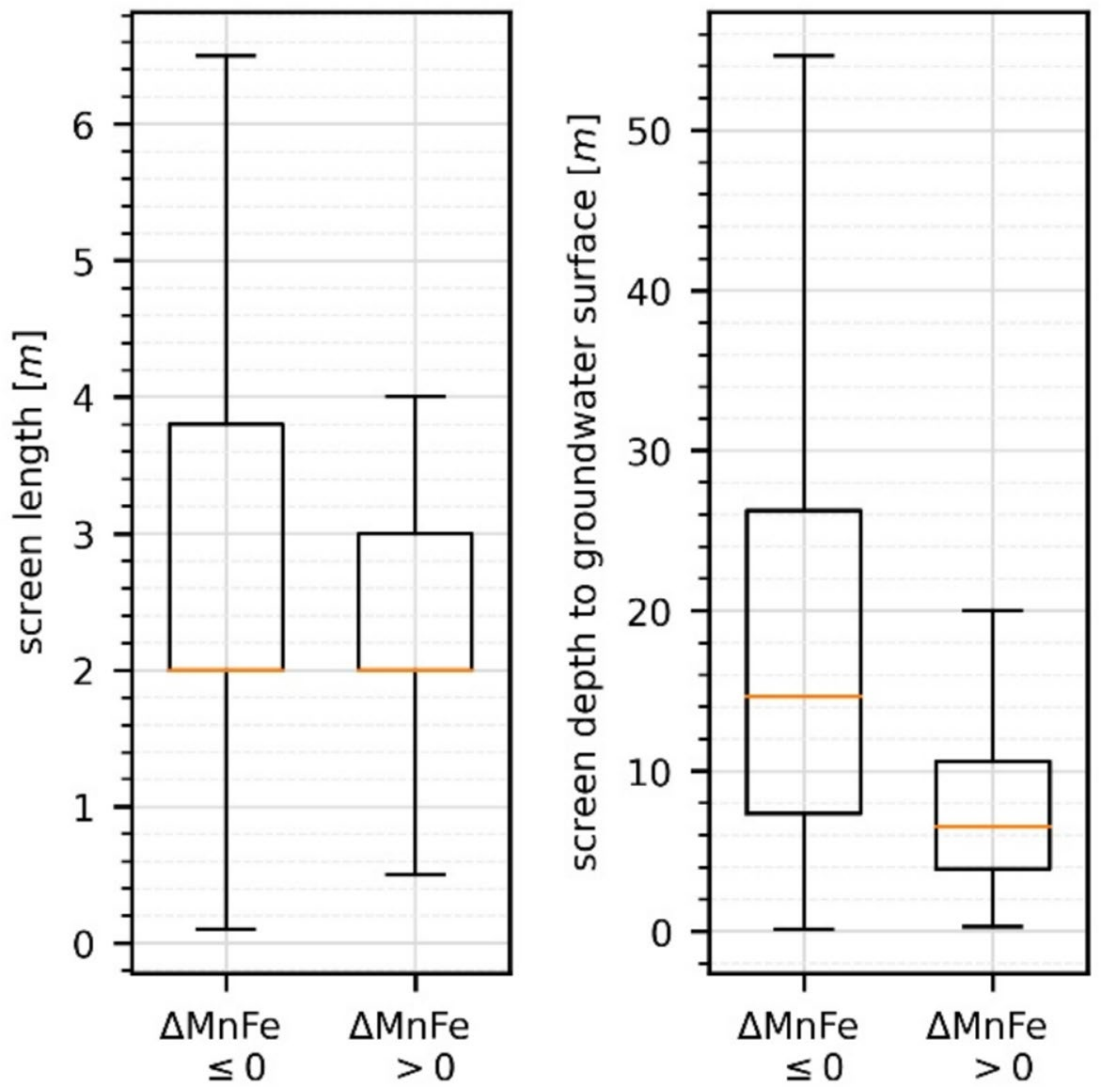
Δ_Mn-Fe_ values ≤ 0 and Δ_Mn-Fe_ > 0 opposed to screen length and screen depth to the groundwater surface. While positive Δ_Mn-Fe_ values were present in median depth (brown line in the graphs) of 6 m below the water table, negative values were sampled at a median depth of 14.5 m below the groundwater table

A total of 309 of the 1872 (17%) wells representing recent groundwater compositions had positive Δ_Mn-Fe_ values (Fig. 7a). They were mostly located in the porous aquifers of the North German Plain. Most of the positive Δ_Mn-Fe_ values (65%) originated from the geests, followed by 27% located in the fluviatile lowlands. With respect to depth, positive Δ_Mn-Fe_ values were observed at significantly lower depths to the groundwater surface, with a median of 6.5 m compared to samples with Δ_Mn-Fe_ ≤ 0 at a median of 14.6 m below the water table (Wilcoxon rank sum *p* = 1.39e−25 < 0.05, *U* = 10.45) (Fig. 4). There was no statistical evidence that the screen length of monitoring wells was correlated with whether Δ_Mn-Fe_ was positive or negative (Wilcoxon rank sum *p* > 0.05).

**Fig. 5:**
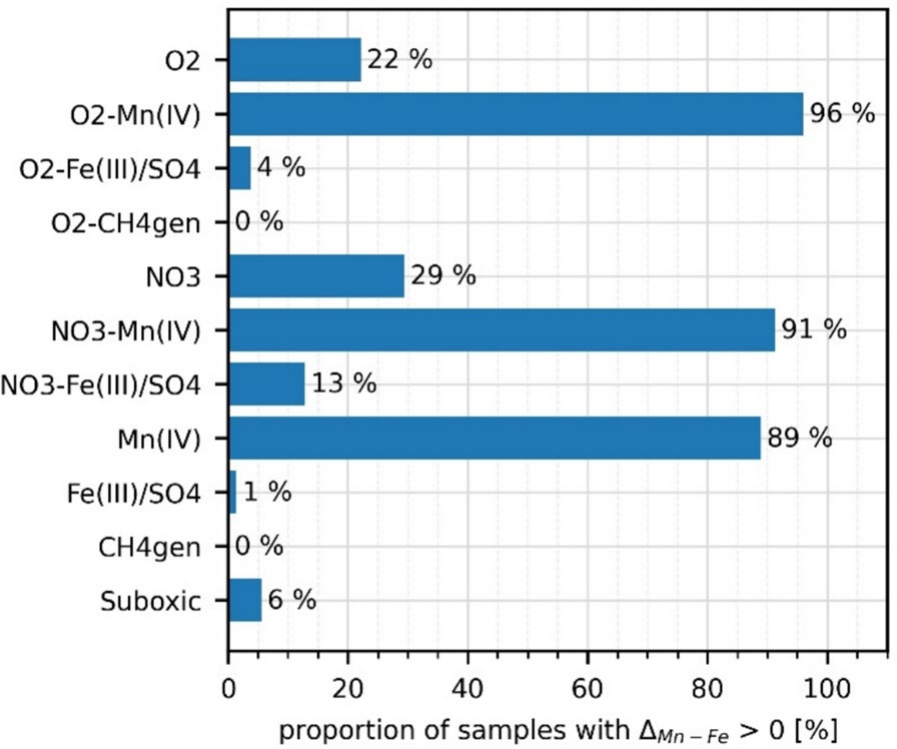
Proportion of classified redox processes in monitoring wells with a positive Δ_Mn-Fe_ (n_total_ = 309)

Most of the positive Δ_Mn-Fe_ values were found in samples taken under redox conditions influenced by manganese reduction (> 89%) (Fig. 5).

### Time series data

**Fig. 6:**
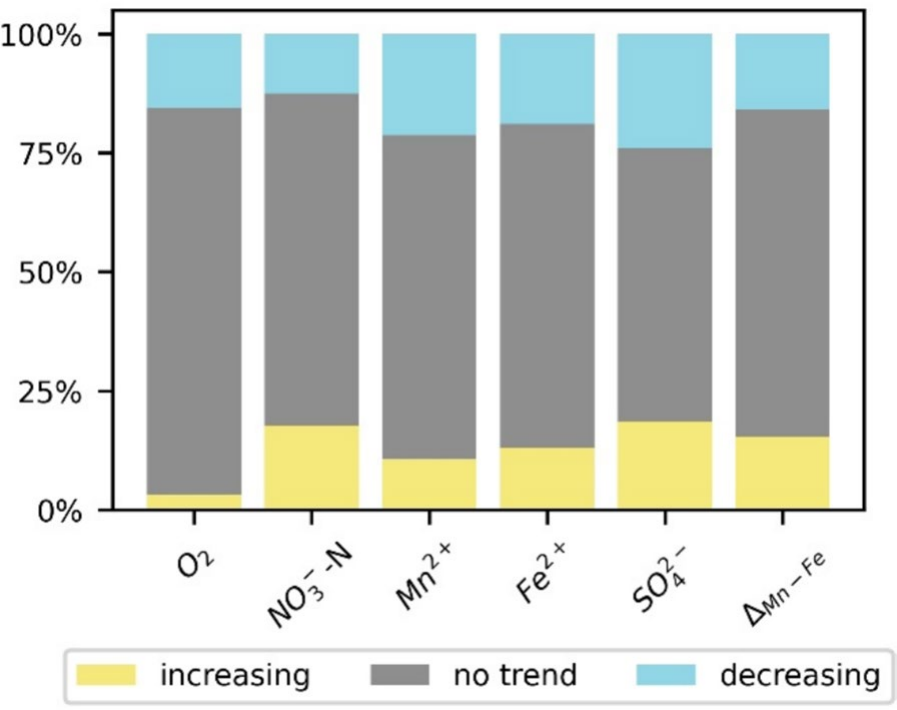
The Mann-Kendall trend test results for O_2_, NO_3_-N, Mn^2+^, Fe^2+^, and SO_4_^2−^ as well as the parameter Δ_Mn-Fe_. Most of the wells exhibited redox conditions without trends

Overall, 1024 monitoring wells with a total of 16,072 samples contributed to the time series analyses. The majority of the Δ_Mn-Fe_ values (69%) remained stable in accordance with the results of the Mann-Kendall trend test, whereas 15% showed increasing and 16% decreasing trends, respectively. This percentage of trends in the Δ_Mn-Fe_ values coincides with the number of trends observed for O_2_, NO_3_^−^-N, Mn^2+^, Fe^2+^, and SO_4_^2−^. They remained stable in 57% of the wells for SO_4_^2−^ to 81% for O_2_. While NO_3_^−^-N tended to increase, O_2_, Mn^2+^, Fe^2+^, and SO_4_^2−^ concentrations tended to decrease more often (Fig. 6).

**Fig. 7:**
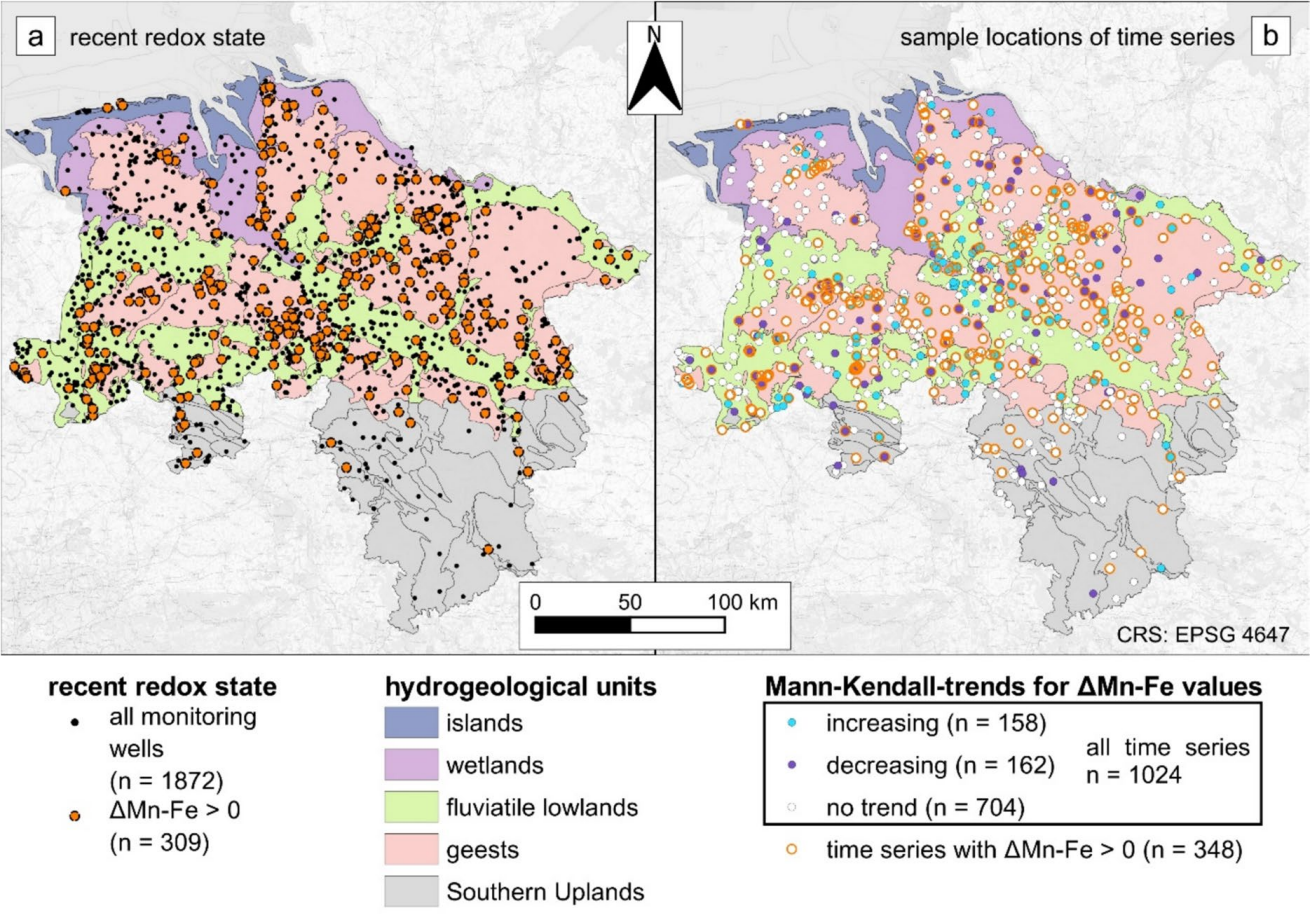
Monitoring wells of the recent redox conditions, with those highlighted in red having a Δ_Mn-Fe_ > 0 (**a**). Location of monitoring wells for trend analysis with the Mann-Kendall trend test results for the parameter Δ_Mn-Fe_ (**b**). Most Δ_Mn-Fe_ > 0 and time series including at least one positive Δ_Mn-Fe_ value were located in the geests (**a**, **b**)

Wells with at least one positive Δ_Mn-Fe_ value showed a spatial distribution similar to that of the current redox conditions (Fig. 7): two-thirds (64%) of the time series with at least one positive Δ_Mn-Fe_ value were located in geests, followed by the lowlands (Fig. 7b).

## Discussion

Surveillance monitoring of shallow groundwater should be designed to describe the composition of groundwater, to detect trends in parameters, and to control the effectiveness of measures to improve the quality of the water. This study discusses problems in assessing groundwater composition at a regional scale due to different redox conditions and offers an approach for managing these limitations.

### Trends and Δ_Mn-Fe_

**Fig. 8:**
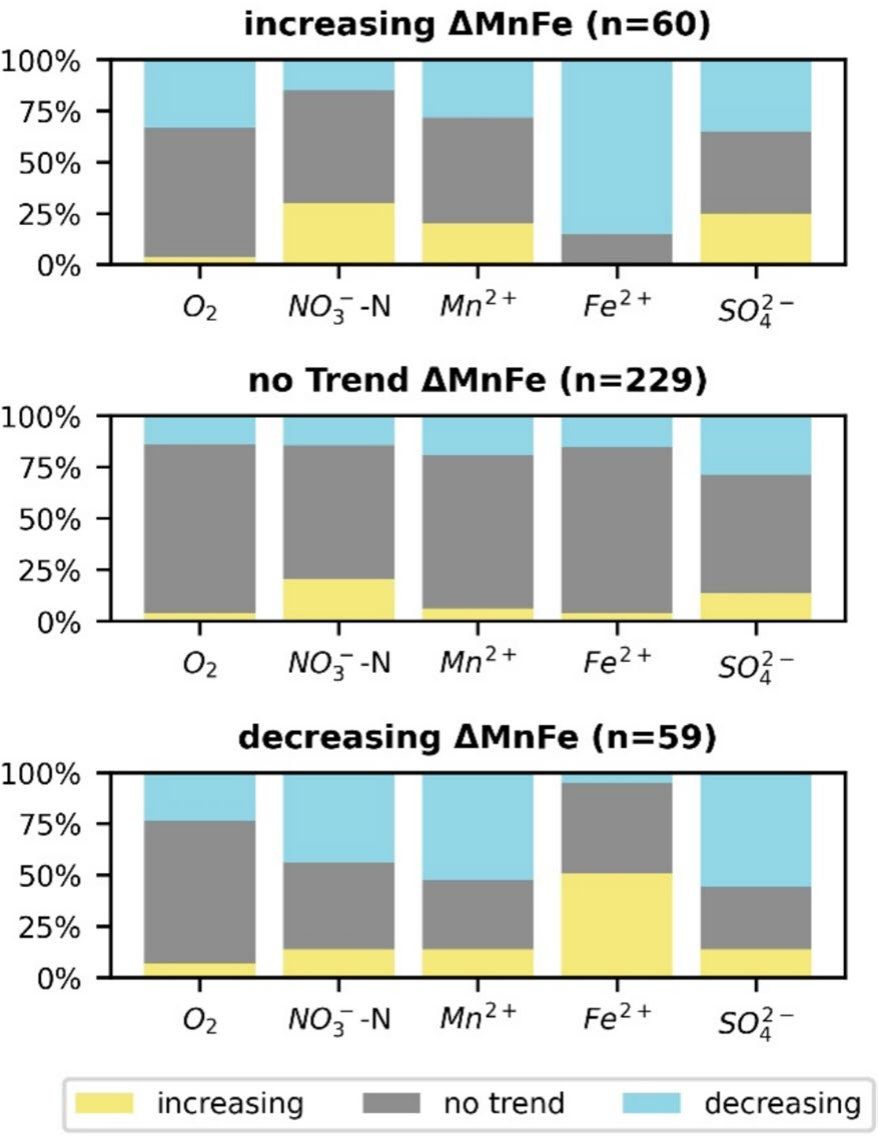
Trends of O_2_-, NO_3_^−^-N, Mn^2+^, Fe^2+^, and SO_4_^2−^ concentrations for monitoring wells separated into groups showing increasing, decreasing, or no trends with respect to the parameter Δ_Mn-Fe_

Within the study area, the Δ_Mn-Fe_ value was applied to characterize redox conditions in addition to O_2_, NO_3_^−^, Mn^2+^, Fe^2+^, and SO_4_^2−^. Up to 30% of the monitoring wells exhibited trends for many parameters sensitive to redox conditions (Fig. 8). This percentage is relevant since components such as cadmium (Kubier & Pichler, 2019) or uranium (Riedel & Kübeck, 2018) might be mobilized from the aquifer into groundwater if redox conditions change. In some studies, such a change in redox conditions in groundwater was interpreted as an effect of diffuse input of nitrate from agricultural areas, with nitrate functioning as an oxidizing agent in the aquifer (e.g., Houben et al., 2017; Kubier et al., 2019; Riedel & Kübeck, 2018).

Koopmann et al. (2020) revealed that a detailed look at dissolved Fe and Mn concentrations could help to identify the input of nitrate to shallow groundwater. These authors simulated the occurrence of redox environments at the laboratory scale and could show that Mn^2+^ would remain stable in the presence of increasing NO_3_^−^ concentrations as long as the NO_3_^−^ surplus was consumed to oxidize Fe^2+^ (Eq. 1).

1 $$\mathrm{NO}_3^-\:+\:5\mathrm{Fe}^{2+}+\:7{\mathrm H}_2\mathrm O\:=\:0.5{\mathrm N}_2+5\mathrm{FeOOH}+9\mathrm H^+$$

**Fig. 9:**
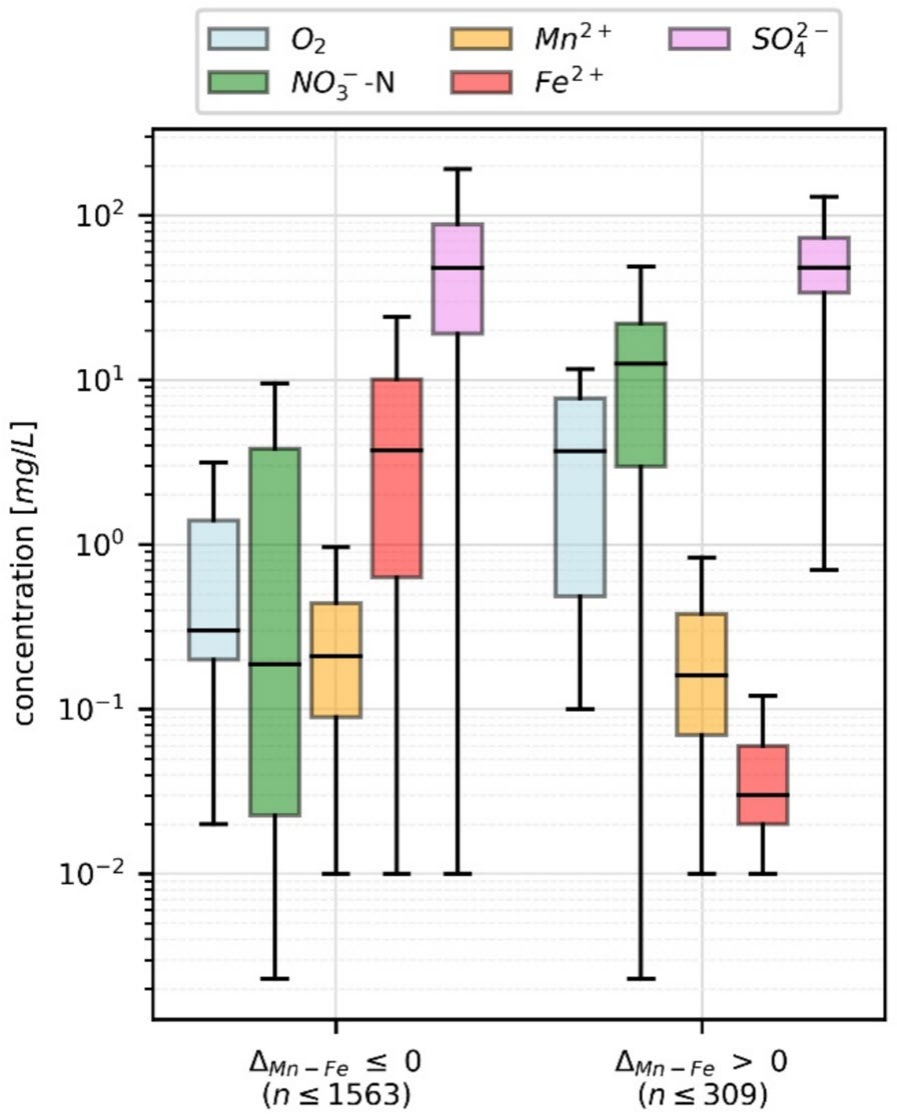
Range of O_2_-, NO_3_^−^-N, Mn^2+^, Fe^2+^, and SO_4_^2−^ concentrations compared for monitoring wells divided into two classes with Δ_Mn-Fe_ > 0 and Δ_Mn-Fe_ ≤ 0. The group with Δ_Mn-Fe_ > 0 is characterized by Fe^2+^ concentration of more than an order of magnitude below those with an Δ_Mn-Fe_ ≤ 0. The nitrate concentrations were antagonistic, whereas the Mn^2+^ concentration remained unaffected

Consequently, the Δ_Mn-Fe_ values became positive. Such changes in the composition of shallow groundwater were also observed in the study area (Figs. 6 and 8). Subdividing the monitoring wells into two groups with Δ_Mn-Fe_ > 0 and < 0 revealed that, in both groups, the Mn^2+^ concentrations were within the same range (Wilcoxon rank sum *p* > 0.05), whereas NO_3_^−^ showed higher and Fe^2+^ concentrations were lower in the group with Δ_Mn-Fe_ > 0 (Wilcoxon rank sum *p* < 0.05) (Fig. 9). Accordingly, the assessment of field data in this study supported the observations at the laboratory scale of Koopmann et al. (2020), and the parameter Δ_Mn-Fe_ > 0 could be considered an appropriate proxy for nitrate reduction in groundwater.

**Fig. 10:**
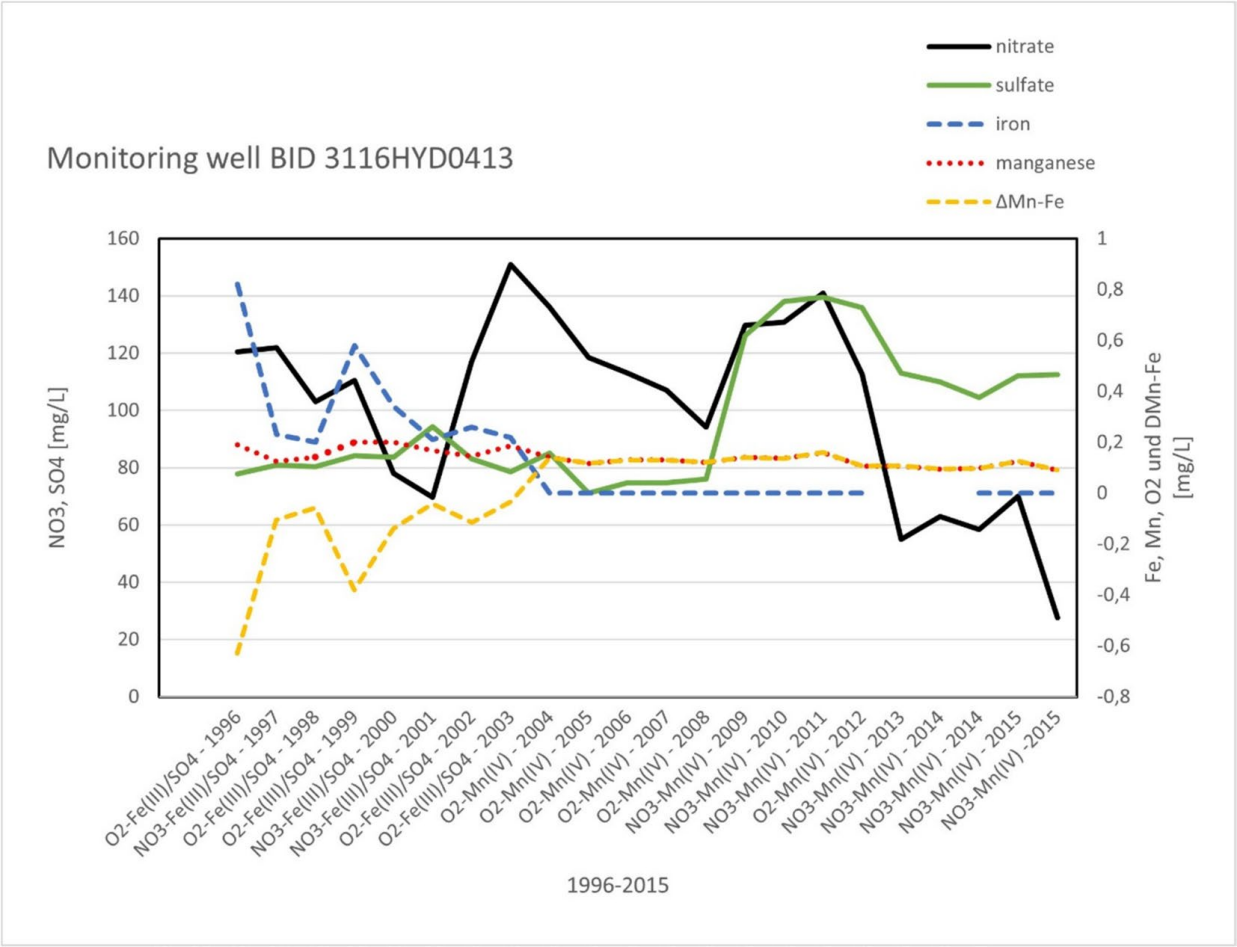
Starting in 1996, the groundwater composition at monitoring well BID 3116HY0413 changed until 2015. The screen had a length of 3 m and was positioned 27 m below ground level. In 1996, the processes of iron and sulfate reduction were dominating and then disappeared in 2003 in favor of manganese reduction and denitrification

**Fig. 11:**
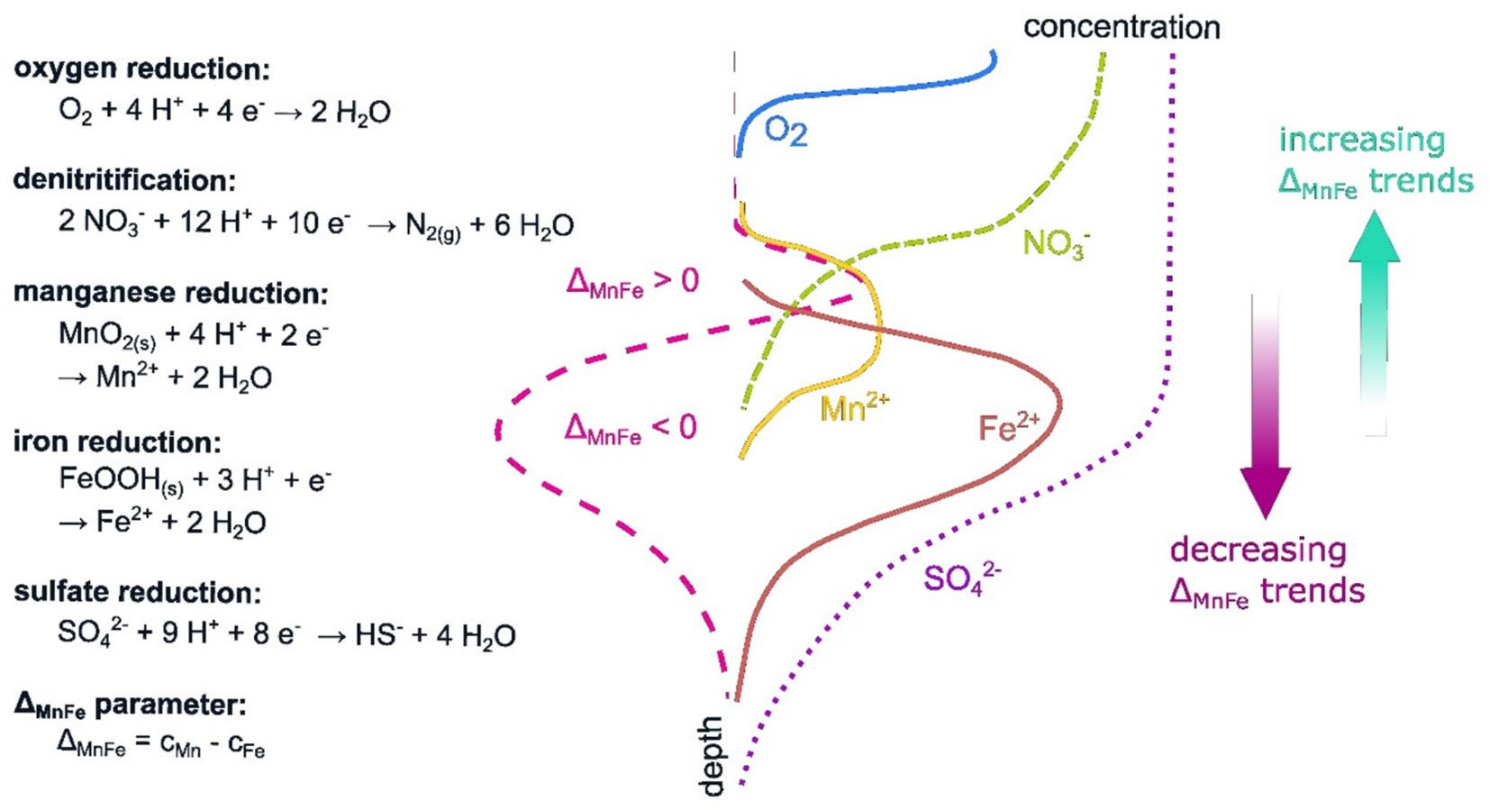
Vertically oriented sequence of redox reactions from the groundwater surface to depth in a recharge area

The reasons for such changes in redox parameters were analyzed separately for the different wells. As an example, monitoring well 3116HY0413 (Fig. 10) revealed that the NO_3_^−^ concentration in 1996 exceeded 100 mg/L. At the same time, Fe^2+^ was measured close to 1 mg/L, and until 2003, it decreased below the detection limit. Meanwhile, Mn^2+^ remained stable. In parallel, the parameter Δ_Mn-Fe_ turned positive. According to the classification of Jurgens et al. (2009), in 1996, the processes of iron and sulfate reduction were dominating and then disappeared in 2003 in favor of manganese reduction and denitrification. The well was situated in an area with a high fraction of arable land and with groundwater recharge exceeding 200 mm/a. In such areas, a diffuse input of nitrate with sewage water and a vertically oriented sequence of redox processes could be expected. Assuming a vertically oriented sequence of redox processes (Fig. 11), the decreasing Fe^2+^ might indicate that a permanent input of NO_3_^−^ with the sewage water consumed the Fe^2+^ and the sequence of redox conditions moved downward with time.

Houben et al. (2017) computed such a vertical movement of redox conditions for a comparable site in North Germany and estimated an annual average shift in the NO_3_^−^ reduction front of less than 1 cm/a. This movement was dependent on the NO_3_^−^concentration in the sewage water, the recharge rate, and the denitrification capacity in the aquifer. In the end, such a downward propagation of redox zones is not easy to detect because, along the length of a screen, different redox conditions are sampled simultaneously and mixed. But the example of monitoring well 3316HYD0413 made obvious that Δ_Mn-Fe_ turned positive when iron reduction faded out in 2004, while manganese reduction became the dominant redox process. Therefore, the example showed that the parameter Δ_Mn-Fe_ could serve as a helpful proxy to identify such a change in the redox conditions in a monitoring well.

### Monitoring network to describe the composition of shallow groundwater

**Fig. 12:**
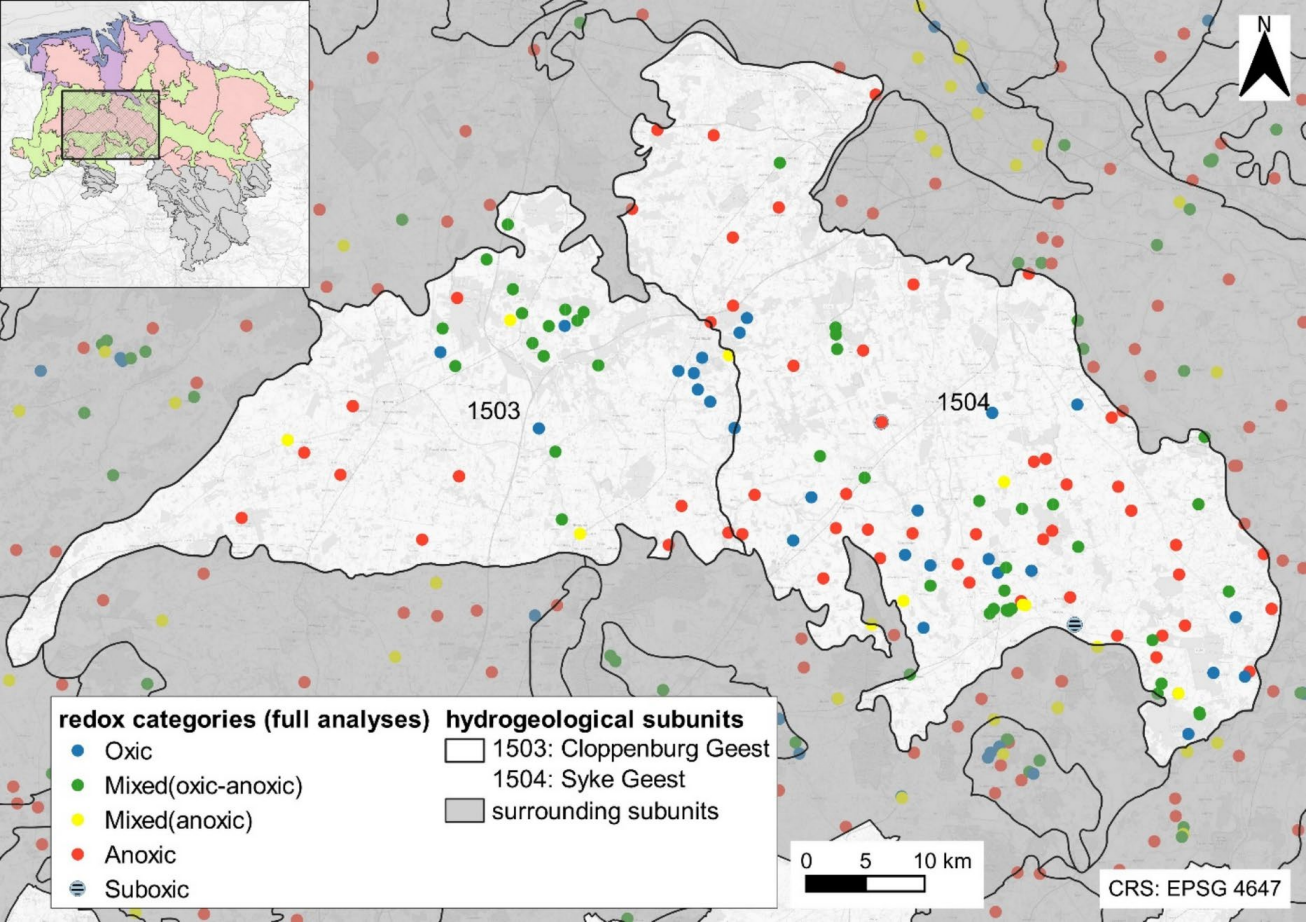
Redox categories in the hydrogeological subunits Cloppenburg and Syke Geest, both situated in the western part of the study area (see small map). Here, the redox conditions differ over short distances between monitoring wells

Factors influencing the composition of shallow groundwater in addition to redox conditions are the depth and length of well screens, the groundwater recharge rate, and land usage in the catchment area. This variety of items often raised the wish of simplification if large data sets should be interpreted. Accordingly, in Lower Saxony, areas with homogeneous hydrogeological properties were defined, so-called hydrogeological units with subunits (Elbracht et al., 2016). However, despite similar geological characteristics, the composition of groundwater varied even within such apparent homogenous units, e.g., in the geest areas of Cloppenburg and Syke (Fig. 12).

To analyze such ambiguous parameter distributions in groundwater, in many recent studies, statistical methods were applied. For example, Kiecak et al. (2023) provided a geochemical characterization of shallow groundwater in Munich, Germany, on the basis of multivariate statistics. The authors identified mineral dissolution, salinization, and redox reactions as the processes involved in the development of hydrogeochemical facies.

In further studies, Knoll et al. (2019, 2020) tested the potential of the machine learning technique of random forest (RF) and quantile random forest (QRF) to estimate the spatial distribution of nitrate concentrations in groundwater in Germany, especially in Hessen. These authors noted that the dominant factors controlling the nitrate concentration were the redox conditions in the groundwater body, the hydrogeological units, and the percentage of arable land.

**Fig. 13:**
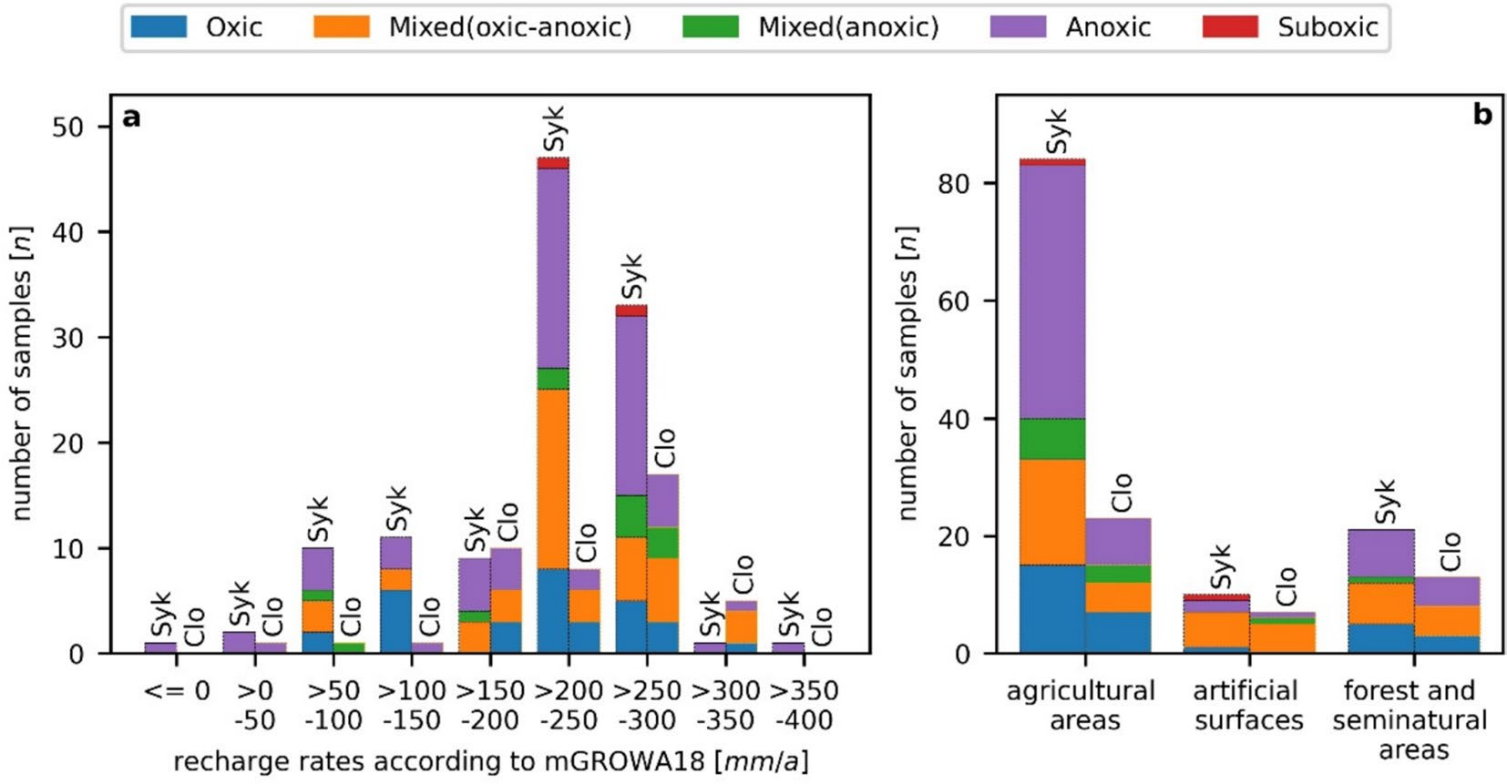
**a** Redox categories in classes of recharge for the hydrogeological subunits Syke (Syk) and Cloppenburg (Clo) geests. **b** Distribution of redox categories of the Syke (Syk) and Cloppenburg (Clo) geest with respect to land use classes after the CORINE land cover system (BKG 2018)

However, these conclusions do not seem to explain the spatial distribution of redox conditions in groundwater of the geest in our study area because these hydrogeological subunits were considered homogenous with respect to the aquifer, usage, and recharge rates. In fact, the redox conditions in the geest subunits of Cloppenburg and Syke seemed to be independent of these factors: Different redox conditions could be found in any type of usage or class of recharge (Fig. 13).

**Fig. 14:**
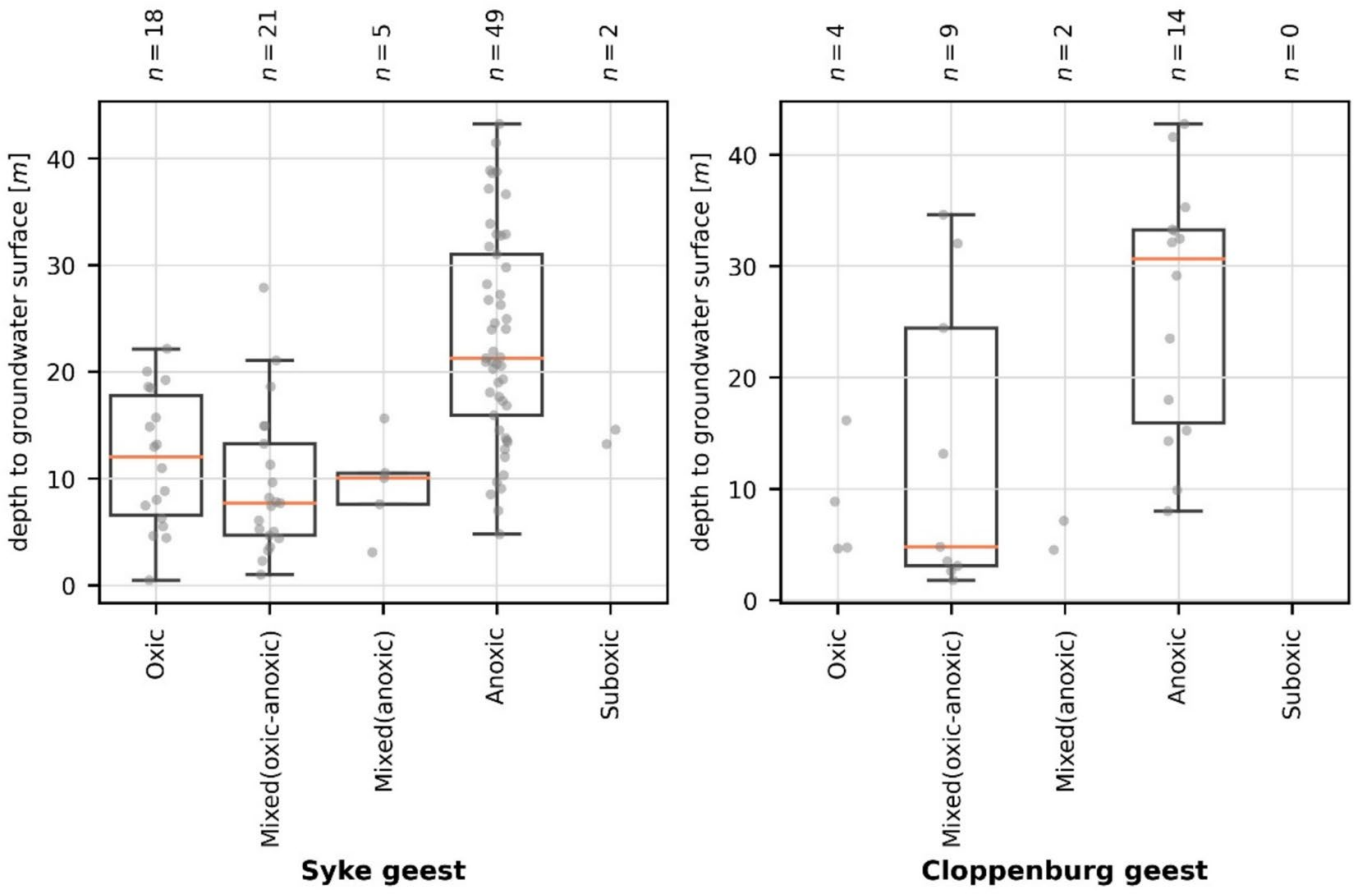
Redox category of groundwater in the hydrogeological subunits Syke and Cloppenburg geest opposed to the depth of the screen to the groundwater surface

Another factor potentially influencing the measured groundwater composition might be screen length since long screens can lead to the mixing of water with different redox conditions during sampling (e.g., Hamer et al., 2020; McMahon & Chapelle, 2008). However, in the study area, most of the wells had similar screen lengths of 2 m (Table 3). In the end, the depth of the screen below the water table was more relevant. Accordingly, in the geest units of Cloppenburg and Syke, often anoxic groundwaters were sampled if the screens were at least more than 20 m below the groundwater table, whereas when they were closer than 20 m to the water table, oxic conditions were often observed (Fig. 14). This coincides with the cascade of redox reactions oriented along the flow direction and follows the conceptual model mentioned above (Fig. 11).

Assuming such as depth dependence for the occurrence of redox conditions, Wriedt et al. (2019) classified the monitoring wells in our study area into depth classes and applied kriging to regionalize nitrate concentrations on the basis of semivariograms. However, the calculated geostatistical relation between the distance among monitoring wells and their variance in NO_3_^−^ concentrations showed no correlation or a weak correlation for deep screens ranging between 30 and 60 m below the groundwater surface. In shallow groundwater at distances < 30 m from the groundwater table, no correlation was observed. From a statistical perspective, the lacking mathematical relation among redox-sensitive parameters such as NO_3_^−^ and the distance between monitoring wells in shallow groundwater indicates that redox conditions can vary on a scale smaller than the distance between monitoring wells. Consequently, a mathematical interpolation based on a distance-dependent function is not adequate.

One reason is the sedimentology of the porous aquifers in the study area, which were often deposited in glacial environments. In such a glacial facies, Hansen et al., (2014) calculated the depth of the redox interface in a water-saturated till in Denmark on the basis of samples that had been analyzed to estimate recharge rates for oxic water through the till and oxygen reducing capacities within the till. The authors observed a mismatch between the depth of the redox interface found in the field and the calculated depth. These authors suggested that the mismatch was the result of sedimentological heterogeneity. Such heterogeneity could also be expected in the glacifluvial sediments of the aquifers in this study area because in the North German Plain, the shallow groundwater was sampled within Holocene and Pleistocene porous aquifers, mostly of fluvial or glaciofluvial origin (Elbracht et al., 2016). In such sediments, the grain size distribution differs over short distances, and consequently, the effective porosity (Meyer et al., 2018) and hydraulic conductivity in the aquifer also differ. Like the grain size distribution, other components in an aquifer, such as pyrite (Houben et al., 2017) or organic matter (Wisotzky et al., 2018), might also vary. This typical heterogeneity is the result of facies changes due to fluctuating flow energy during the sedimentation of the aquifer (e.g., Lesemann et al., 2014).

In the end, the sedimentological heterogeneity of aquifers is responsible for anisotropy, hydraulic conductivity ranging in order of magnitude, and ranges in the contents of organic matter, manganese and iron oxides and sulfides, all chemically functioning as oxidizing or reducing capacities (Christensen et al., 2000). This combination of items explains why, in the groundwater of porous glacial aquifers, the redox conditions can vary on a range far below the lateral distance between monitoring wells in a typical network for groundwater monitoring.

### Implications for assessment and groundwater protection measures

Given the spatial and temporal variability of redox conditions at scales below the distance of monitoring wells, questions arise regarding how to assess groundwater distinctly and how to design clear measures to control or improve the composition of shallow groundwater. Recently, in the study area, the responsible authorities determined areas where nitrate fertilizer application should be restricted because in the respective area the threshold value and further criteria for nitrate in shallow groundwater were exceeded. However, this measure recently failed at the authorized court (Oberverwaltungsgericht OVG, 2025). One reason was that it seemed to be not logical to have both, on the one hand, monitoring wells exceeding threshold values and, on the other hand, direct neighborhood wells without or with low nitrate in such areas. Following the arguments for that judgment, this regional implementation of the European Water Framework (2000/60/EC) and Nitrate Directive (1991/676/EEC) did not allow a doubtless interpretation of the groundwater composition here with respect to the redox-sensitive concentration of nitrate.

Many authors have discussed this problem and concluded that more monitoring wells are necessary (e.g., Wolters et al., 2022), especially those with short screens (Hansen et al., 2011) or multilevel screens (Lasagna & De Luca, 2016). Considering that the number of existing monitoring wells in the study area exceeds 5000 piezometers, more wells are not a realistic option.

In the example of the geest units Cloppenburg and Syke (Fig. 12), 36 of overall 94 monitoring wells exceeded the nitrate-threshold of 50 mg/L. None of the wells with elevated nitrate concentrations had anoxic conditions. When all anoxic and mixed wells with anoxic redox conditions were skipped, 21 wells with oxic conditions would remain and within that group of monitoring wells with oxic conditions, two-thirds of the wells showed too high nitrate concentrations (Table 4).

**Table 4:** Redox conditions and nitrate concentrations in the geests of Cloppenburg and Syke

Redox condition (classified acc. to Jurgens et al. (2009)	Number of wells	NO_3_ > 50 mg/L	NO_3_ < 50 mg/L
Oxic	22	13	9
Mixed oxic-anoxic	41	20	21
Mixed anoxic	6	3	3
Anoxic	25	0	25
(All monitoring wells)	94	36	58

Consequently, if monitoring is intended to assess emissions, only wells where emissions to groundwater are not altered by redox processes should be considered.

## Conclusion

Redox processes complicate groundwater monitoring, since redox conditions vary on scales below the distance among the wells in a monitoring network, even within areas that are assumed to be homogeneous in terms of recharge rate, usage, and hydrogeology. Additionally, the application of statistical methods to describe the chemical composition of groundwater spatially is often confined, since many of those methods assume isotropy and homogeneity at a spatial resolution, which is not given in fluviatile or glaciofluviatile aquifers. Consequently, some authors have concluded that more monitoring wells are necessary, best with short screens. At first glance, that is a desired option, but as far as surveillance monitoring, especially in recharge areas, is concerned, the authors of this study recommend considering fewer wells, depending on the question of interest, and applying a 3-step approach. In the first step, the monitoring wells should be classified with respect to redox conditions, as conducted in this study. After assorting the monitoring wells this way, in a second step, wells with oxic conditions can reflect the input of chemicals to groundwater via sewage water. These monitoring wells are candidates for controlling diffuse input via sewage water and for checking the efficiency of measures to improve or protect groundwater. Assessing monitoring wells with anoxic conditions is a subordinated priority for this kind of question. On the other hand, these anoxic wells allow one to see the result of reducing capacities of the aquifer, such as pyrite or organic matter, which can buffer the input of potentially oxidizing agents such as NO_3_^−^ from recharge water. The third step is to apply the parameter ∆_Mn-Fe_ combined with trend tests because ∆_Mn-Fe_ is sensitive to oxidizing emissions into groundwater and can serve as a proxy for potential changes in redoxcline, e.g., due to nitrate emissions to shallow groundwater.

This sequence of data evaluation is helpful for identifying wells in the same redox state as a prerequisite for an assessment and for observing changes in the groundwater composition of shallow groundwater even if different redox conditions are present.

## Data Availability

No datasets were generated or analysed during the current study.

## References

[CR1] Appelo, C. A. J., & Postma, D. (2005). *Geochemistry, groundwater and pollution* (p. 649). Balkema Publishers.

[CR2] Blicher-Mathiesen, G., McCarty, G. W., & Nielsen, L. P. (1998). Denitrification and degassing in groundwater estimated from dissolved dinitrogen and argon. *Journal of Hydrology,**208*, 16–24.

[CR3] Bundesamt für Kartographie und Geodäsie BKG (2018). *CORINE Land Cover 5 ha CLC5 *(p*. *7) . report. Download at: https://sg.geodatenzentrum.de/web_public/gdz/dokumenta-tion/deu/clc5_2018.pdf. Accessed 3 Sept 2025

[CR4] Christensen, T. H., Bjerg, P. L., Banwart, S. A., Jakobsen, R., Heron, G., & Albrechtsen, T. J. (2000). Characterization of redox conditions in groundwater contaminant plumes. *Journal of Contaminant Hydrology,**45*, 165–241.

[CR5] Collins, S. B., Singh, R., Mead, S. R., et al. (2025). Assessment of spatial variability and temporal stability of groundwater redox conditions in New Zealand. *Environmental Monitoring and Assessment,**197*, 58. 10.1007/s10661-024-13427-y 10.1007/s10661-024-13427-y 39680250

[CR6] Deutsches Institut for Normung (DIN) (2018): *General requirements for the competence of testing and calibration laboratories* (ISO/IEC 17025:2017); 65 pp. Beuth Verlag, Berlin; 10.31030/2731745

[CR7] Elbracht, J., Meyer, R., & Reutter, E. (2016). Hydrogeological areas and subareas in Lower Saxony. *GeoBericht,**3*, 3–118.

[CR8] Erickson, M. L., Elliott, S. M., Brown, C. J., Stackelberg, P. E., Ransom, K. M., Reddy, J. E., & Cravotta, C. E., III. (2021). Machine-learning predictions of high arsenic and high manganese at drinking water depths of the Glacial Aquifer System, Northern Continental United States. *Environmental Science and Technology,**55*(9), 5791–5805. 10.1021/acs.est.0c06740 33822585 10.1021/acs.est.0c06740

[CR9] Ertl, G., Bug, J., Elbracht, J., Engel, N., & Herrmann, F. (2019). Grundwasserneubildung von Niedersachsen und Bremen -Berechnungen mit dem Wasserhaushaltsmodell mGROWA18. *GeoBer, 36, *3–54.

[CR10] Eschenbach, W., Budziak, D., & Elbracht, J. (2018). Möglichkeiten und Grenzen der Validierung flächenhaft modellierter Nitrateinträge ins Grundwasser mit der N_2_/Ar-Methode. *Grundwasser,**23*, 125–139. 10.1007/s00767-018-0391-6

[CR11] European Commission. (1991). Council Directive 91/676/EEC of 12 December 1991 concerning the protection of waters against pollution caused by nitrates from agricultural sources. *Official Journal of European Communities L 375, *1–8.

[CR12] European Commission. (2000). Directive 2000/60/EC of the European Parliament and of the Council of 23 October 2000; Establishing a framework for community action in the field of water policy; 2000/60/EC. *Official Journal of European Communities L 327,* 1–72.

[CR13] European Commission. (2006). Directive 2006/118/EC of the European parliament and of the council of 12 December 2006 on the protection of groundwater against pollution and deterioration; 2006/118/EC. *Official Journal of the European Communities L 372. *19–31*.*

[CR14] Gran, G., Johansson, A., & Johansson, S. (1981). Automatic titration by stepwise addition of equal volumes of titrant part VII. Potentiometric precipitation titrations. *The Analyst,**106*, 1109–1118.

[CR15] Hamer, K., Gudenschwager, I., & Pichler, T. (2020). Manganese (Mn) concentrations and the Mn-Fe relationship in shallow groundwater: Implications for groundwater monitoring. *Soil Systems,**4*, 49–19. 10.3390/soilsystems4030049

[CR16] Hansen, A. L., Christensen, B. S. B., Ernstsen, V., He, X., & Refsgaard, J. S. (2014). A concept for estimating depth of the redox interface for catchment-scale nitrate modelling in a till area in Denmark. *Hydrogeology Journal,**22*, 1639–1655. 10.1007/s10040-014-1152-y

[CR17] Hansen, J. R., Ernstsen, V., Refsgaard, J. C., & Hansen, S. (2008). Field scale heterogeneity of redox conditions in till-upscaling to a catchment nitrate model. *Hydrogeology Journal,**16*, 1251–1266. 10.1007/s10040-008-0330-1

[CR18] Hansen, B., Thorling, L., Dargaard, T. & Erlandsen, M. (2011). Trend reversal of nitrate in Danish groundwater - a reflection of agricultural practices and nitrogen surpluses since 1950. *Environmental Science & Technology*, *45*, 228–234. 10.1021/es102334u 10.1021/es102334u 21138289

[CR19] Helsel, D., Hirsch, R. M., Ryberg, K. R., Archfeld, S. A., & Gilroy, E. J. (2020). Statistical methods in water resources. In: *U.S. Geological Survey Techniques and Methods*, Book 4, U.S. Geological Service, chap A3, p 458

[CR20] Houben, G. H., Sitnikova, M. A., & Post, V. E. A. (2017). Terrestrial sedimentary pyrites as a potential source of trace metal release to groundwater e a case study from the Emsland, Germany. *Appl. Geochem.,**76*, 99–111.

[CR21] Hunter, J. D. (2007). Matplotlib - a 2D graphics environment. *Computing in Science & Engineering,**9*(3), 90–95.

[CR22] Hussain, M. M., & Mahmud, I. (2019). PyMannKendall – A Python package for non-parametric Mann Kendall family of trend tests. *Journal of Open Source Software*. 10.21105/joss.01556

[CR23] Jurgens, B. C., McMahon, P. B., Chapelle, F. H., & Eberts, S. M. (2009). An excel® workbook for identifying redox processes in ground water. *US Geol. Surv. Open File Rep*. 2009, 8. Available online: http://pubs.usgs.gov/of/2009/1004. Accessed on 30 July 2020.

[CR24] Kendall, M. G. (1975). *Rank correlation methods*. Charles Griffin.

[CR25] Kiecak, A., Huch, J., Albarrán-Ordás, A., Chavez-Kus, L., & Zosseder, K. (2023). Interpretation of hydrogeochemistry of the Upper Freshwater Molasse (Obere Süßwassermolasse) in the Munich area (Bavaria, Germany) using multivariate analysis and three-dimensional geological modelling. *Hydrogeology Journal,**32*, 891–912. 10.1007/s10040-023-02761-z

[CR26] Knoll, L., Breuer, L., & Bach, M. (2019). Large scale prediction of groundwater nitrate concentrations from spatial data using machine learning. *Science of the Total Environment,**668*, 1317–1327. 31018471 10.1016/j.scitotenv.2019.03.045

[CR27] Knoll, L., Breuer, L., & Bach, M. (2020). Nation-wide estimation of groundwater redox conditions and nitrate concentrations through machine learning. *Environmental Research Letters,**15*, 064004. 10.1088/1748-9326/ab7d5c

[CR28] Koopmann, S., Fröllje, H., Hamer, K., Kubier, A., & Pichler, T. (2020). Eisen-Mangan-Anomalien im Grundwasser—Analyse der beeinflussenden Prozesse (Fe-Mn-anomalies of groundwater—Analysis of influencing processes). *Grundwasser,**25*, 113–126.

[CR29] Kruskal, W. H., & Wallis, W. W. (1952). Use of ranks in one-criterion variance analysis. *Journal of American Statistical Association,** 47*, 583–621. 10.1080/01621459.1952.10483441

[CR30] Kubier, A., Hamer, K., & Pichler, T. (2019). Cadmium background levels in groundwater in an area dominated by agriculture. *Integrated Environmental Assessment and Management,**16*, 103–113. 10.1002/ieam.4198 31368630 10.1002/ieam.4198

[CR31] Kubier, A., & Pichler, T. (2019). Cadmium in groundwater − A synopsis based on a large hydrogeochemical data set. *Science of the Total Environment,**689*, 831–842. 31280165 10.1016/j.scitotenv.2019.06.499

[CR32] Lasagna, M., & De Luca, D. A. (2016). The use of multilevel sampling techniques for determining shallow aquifer nitrate profiles. *Environmental Science and Pollution Research,**23*, 20431–20448. 10.1007/s11356-016-7264-2 27460024 10.1007/s11356-016-7264-2

[CR33] Lesemann, J. E., Piotrowski, J. A., & Wysota, W. (2014). Genesis of the ‘glacial curvilineation’ landscape by meltwater processes under the former Scandinavian Ice Sheet, Poland. *Sedimentary Geology,**312*(2014), 1–18.

[CR34] Lovley, D. R., & Goodwin, S. (1988). Hydrogen concentrations as an indicator of the predominant terminal electro-accepting reactions in aquatic systems. *Geochimica Et Cosmochimica Acta,**52*, 2993–3003.

[CR35] Mann, H. B. (1945). Non-parametric test against trend. *Econometrica,**13*, 245–259.

[CR36] McMahon, P. B., & Chapelle, F. H. (2008). Redox processes and water quality of selected principal aquifer systems. *Groundwater,**46*, 59–71. 10.1111/j.1745-6584.2007.00385.x 18307432

[CR37] Merz, C., Steidl, J., & Dannowski, R. (2009). Parameterization and regionalization of redox based denitrification for GIS-embedded nitrate transport modeling in Pleistocene aquifer systems. *Environmental Geology,**58*, 1587–1599.

[CR38] Meyer, R., Engesgaard, P., Hinsby, K., Piotrowski, J. A., & Sonnenborg, T. O. (2018). Estimation of effective porosity in large-scale groundwater models by combining particle tracking, auto-calibration and ^14^C dating. *Hydrology and Earth System Sciences,**22*, 4843–4865. 10.5194/hess-22-4843-2018

[CR39] Mouser, P. J., Rizzo, D. M., Rölling, W. F. M., & van Breukelinen, A. M. (2005). A multivariate statistical approach to spatial representation of groundwater contamination using hydrochemistry and microbial community profiles. *Environmental Science and Technology,**39*, 7551–7755. 16245827 10.1021/es0502627

[CR40] Oberverwaltungsgericht Niedersachsen (OVG). (2025). Judgment of the 10^th^ Senate at 28^st^ January 2025 about the determination of areas with restrictions of nitrate fertilizer application; reference number10 KN 66/22. *Die Öffentliche Verwaltung (DÖV).* 577.

[CR41] Ortmeyer, F., Hansen, B., & Banning, A. (2022). Groundwater nitrate problem and countermeasures in strongly affected EU countries—A comparison between Germany, Denmark and Ireland. *Grundwasser*. 10.1007/s00767-022-00530-5

[CR42] Reimann, C., & Filzmoser, P. (2000). Normal and lognormal data distribution in geochemistry: Death of a myth. Consequences for the statistical treatment of geochemical and environmental data. *Environmental Geology,**39*(9), 1001–1014.

[CR43] Riedel, T., & Kübeck, C. (2018). Uranium in groundwater—A synopsis based on a large hydrogeochemical data set. *Water Research,**129*, 29–38. 29127832 10.1016/j.watres.2017.11.001

[CR44] Schafmeister, M. T., Steffen, M., Zeissler, K. O., & Zingelmann, M. (2023). Extension variance: An early geostatistical concept applied to assess nitrate pollution in groundwater. *Hydrogeology Journal,**31*, 1463–1473. 10.1007/s10040-023-02666-x

[CR45] Su, X., Lu, S., & Yuan, W. (2018). Redox zonation for different groundwater flow paths during bank filtration: A case study at Liao River, Shenyang, Northeastern China. *Hydrogeology Journal,**26*, 1573–1589. 10.1007/s10040-018-1759-5

[CR46] Tesoriero, A. J., Terziotti, S., & Abrams, D. B. (2015). Predicting redox conditions in groundwater at a regional scale. *Environmental Science & Technology,**49*, 9657–9664. 26230618 10.1021/acs.est.5b01869

[CR47] Virtanen, P., Gommers, R., Oliphant, T. E., Haberland, M., Reddy, T., Cournapeau, D., Burovski, E., Peterson, P., Weckesser, W., Bright, J., van der Walt, S. J., Brett, M., Wilson, J., Millmann, K. J., Mayorov, N., Nelson, A. R. J., Jones, E., Kern, R., Larson, E., … van Mulbregt, P. (2020). SciPy 1.0: Fundamental algorithms for scientific computing in Python. *Nat. Methods,**17*(3), 261–272. 32015543 10.1038/s41592-019-0686-2 PMC7056644

[CR48] Wisotzky, F., Cremer, N., & Lenk, S. (2018). *Angewandte Grundwasserchemie, Hydrogeologie und hydrogeo-chemische Modellierung*. Springer Verlag Heidelberg.

[CR49] Wolters, T., Bach, T., Eisele, M., Eschenbach, W., Kunkel, R., McNamara, I., Well, R., & Wendland, F. (2022). The derivation of denitrification conditions in groundwater: Combined method approach and application for Germany. *Ecological Indicators*. 10.1016/j.ecolind.2022.109564

[CR50] Wriedt, G., De Vries, D., Eden, T., & Federolf, C. (2019). Regionalisierte Darstellung der Nitratbelastung im Grundwasser Niedersachsens. *Grundwasser,**24*, 27–41. 10.1007/s00767-019-00415-0

[CR51] Wriedt, G., & Randt, C. (2019). Phosphat im Grundwasser Niedersachsens – Verteilung, Einflussfaktoren und Schwellenwert. *Grundwasser,**24*, 109–127. 10.1007/s00767-019-00418-x

[CR52] Yue, S., Pilon, P., & Cavadias, G. (2002). Power of the Mann-Kendall and Spearman’s rhos tests for detecting monotonic trends in hydrological series. *Journal of Hydrology,**259*, 254–271.

